# TME9/379: Development of a WWW Clinical Booking Service

**DOI:** 10.2196/jmir.1.suppl1.e116

**Published:** 1999-09-19

**Authors:** F Sicurello, R Pizzi

**Affiliations:** ^1^Istituto Nazionale Neurologico C. Besta, MilanoItaly

**Keywords:** Telemedicine, Radiology, Intranet, Internet, Images

## Abstract

This application is a hybrid architecture that embeds a previous hospital booking procedure and allows to book visits and diagnostic examinations by remote people, general practitioners, clinical centers via an Internet connection. A PowerBuilder-based Web server has been developed to interface the procedure, in such a way as at any client request, the DDL functions are used through the web server, and at any reply of the Oracle database, a HTML string is generated that goes back to the client. To realize this project many tools have been used:

Internet Information Server - it is a set of applications using dynamic link libraries much faster than the standard CGI ones.

- ASP (Active Server Pages) - this is a new Internet technology that allows to create fine Web pages, platform independent and usable by any browser.
- The VBScript - this is an event-based language, useful to link code to a user interaction or to a system reply.
- HTML - this language describes the structure of a document and defines a set of fixed stiles for the Web pages, and can be read by any editor.
- PowerBuilder Enterprise- it is an object-oriented environment for the development of high-performance client/server and Internet applications on many different OSs.
- SQL*Net - it is an Oracle product that optimizes the network management ensuring interoperability between different computers, OSs, and networks.
- Oracle ODBC32 - it is a standard protocol to access information in SQL databases (Microsoft, Paradox, Oracle, etc.)

The developed software fully manages the hospital booking procedure and is included into the hospital network. In this way the Unique Booking Center can work both inside the hospital and from all over the country by means of a simple Internet connection.

